# Role of damaged mitochondrial transfer in alpha-particle generator ^212^Pb radiation-induced bystander effect

**DOI:** 10.7150/thno.101922

**Published:** 2024-10-14

**Authors:** Mengdie Yang, Lusheng Wang, Shanshan Qin, Xiongxin Dai, Jianguo Li, Liwei An, Lijuan Song, Jie Gao, Zongtai Han, Fei Yu

**Affiliations:** 1 Department of Nuclear Medicine, Shanghai Tenth People's Hospital, Tongji University School of Medicine, Shanghai, China.; 2 Institute of Nuclear Medicine, Tongji University School of Medicine, Shanghai, China.; 3 China Institute for Radiation Protection, Taiyuan, Shanxi, China.; 4 Collaborative Innovation Center of Radiation Medicine of Jiangsu Higher Education Institutions, Suzhou, China.; 5 National Atomic Energy Agency Nuclear Technology (Nonclinical Evaluation of Radiopharmaceuticals) Research and Development Center, Taiyuan, Shanxi, China.; 6 CNNC Key Laboratory on Radio-toxicology and Radiopharmaceutical Preclinical Evaluation, Taiyuan, Shanxi, China.; 7 Department of Stomatology, Shanghai Tenth People's Hospital, Department of Biochemistry and Molecular Biology, Tongji University School of Medicine, 200072, Shanghai, China.

**Keywords:** targeted alpha therapy, lead-212, bismuth-212, radiation-induced bystander effect, mitochondrial transfer

## Abstract

**Rationale:** ^212^Pb, a promising *in vivo* alpha*-*particle generator of ^212^Bi, has aroused much interest as a therapeutic radionuclide. For the development of targeted alpha therapy (TAT), it is important to determine the contribution of targeted effects in irradiated cells, and also of non-targeted effects in non-irradiated bystander cells. Currently, the critical roles of mitochondrial transfer in cellular crosstalk have garnered significant attention. However, the specific involvement of damaged mitochondrial transfer in orchestrating this alpha-particle radiation-induced bystander effect (RIBE) needs to be further explored.

**Methods:** A novel alpha-emitting radiopharmaceutical, ^212^Pb-hydrogel nanoparticles (HNPs), was synthesized and subsequently evaluated its theranostics effects. The impact of irradiated cell-conditioned media (ICCM), collected at different times post-^212^Bi irradiation, on bystander cancer cells regarding cell viability was also investigated. Additionally, damaged mitochondria were isolated and cultured with non-irradiated bystander cells to assess their role.

**Results:** ^212^Pb-HNPs exhibited efficient therapeutic antitumor effects* in vitro*, including increased GSH depletion, ROS accumulation, and mitochondrial damage in irradiated tumor cells. *In vivo* studies demonstrated its imaging potential through SPECT/CT, and RNA sequencing results indicated activation of oxidative stress-related pathways in irradiated tumors. Additionally, ICCM influenced the viability of non-irradiated bystander cells, suggesting a radiation-induced bystander effect by the alpha-particle ^212^Bi. Interestingly, damaged mitochondria isolated from ICCM were observed to enter co-cultured non-irradiated bystander cells. Further experiments confirmed that the transfer of damaged mitochondria results in the death of non-irradiated bystander cells.

**Conclusion:** The present study highlights the theranostic potential of the alpha-particle generator ^212^Pb and, more importantly, elucidates the role of damaged mitochondrial transfer in alpha-particle RIBE. These findings provide a novel theoretical mechanism for the antitumor effects of alpha-particles and expand the clinical application prospects of TAT.

## Introduction

Malignant tumors pose one of the most significant threats to human health, necessitating the development of new precision therapeutic strategies 1. Notably, in recent years, radiopharmaceuticals have emerged as a revolutionary approach for precision oncology due to their superior "efficacy and safety". Historically, the use of beta-particles has been more prevalent both domestically and internationally. However, alpha-particles, with their unique clinical advantages, represent a novel and promising avenue in nuclear medicine for anti-tumor therapy 2, 3. Nevertheless, the biological effects of alpha-particle radiation are complex, and research on the mechanisms of targeted alpha-therapy (TAT) has progressed more slowly than that for beta-particles worldwide. This slow progress has become a critical bottleneck in the clinical translation and application of alpha-particles. Therefore, elucidating the therapeutic mechanisms of alpha-particles is urgently needed to meet the demands of societal development and public health.

One increasingly popular radionuclide is the *in vivo* alpha-particle generator ^212^Pb, which emits 1 (net) alpha-particle and 2 beta-particles within its decay chain. ^212^Pb has a half-life of 10.6 h, extending the time to deliver and target tumors with its short-lived ^212^Bi daughter (T_½_ 60.6 min) 4. This results in a greater therapeutic impact and reduces the dose required for an effective therapeutic benefit. At the same time toxicity to normal tissues is reduced. ^212^Pb possesses several advantages, including independence from reactors and accelerators, high radionuclide purity, well-defined quality control methods, and low long-term bulk supply costs. These attributes make ^212^Pb the most scalable and competitively priced radionuclide for TAT. Additionally, ^212^Pb emits characteristic gamma rays at 238 keV (43.6%), which possess potential for imaging applications 5. Studies using^ 212^Pb showed the feasibility of this isotope in TAT for the treatment of disseminated intraperitoneal disease.

Cellular death is a crucial endpoint that has been extensively studied in cells exposed to radiation. It is essential to assess the contributions of both targeted effects in irradiated cells and non-targeted effects in non-irradiated bystander cells. The transmission of signals between irradiated and non-irradiated cells occurs via gap junctions or soluble signaling mediators. The resultant damage in adjacent non-targeted cells, collectively termed radiation-induced bystander effect (RIBE), includes cell death, chromosomal damage, mutations, and alterations in gene expression and protein levels 6-8. The RIBE was first identified as the phenomenon involving the induction of sister chromatid exchanges by extremely low doses of alpha-particles in non-irradiated cells 9. However, the mechanisms by which alpha particles induce RIBE remain unclear, necessitating further development and refinement of the relevant theoretical framework.

A substantial body of evidence suggests that mitochondria may play a pivotal role in RIBE 10-12. Currently, mitochondrial research has evolved from the "powerhouse" paradigm to the "information processor" paradigm. As the central hub of cellular signal transduction, mitochondria are now recognized as essential signaling entities in the modulation of cellular responses to ionizing radiation. Historically, mitochondrial functions were thought to be confined primarily within the boundaries of the host cell 13-15. However, recent studies have demonstrated that mitochondria and their components can be released into the extracellular space, participating in intercellular communication. This process, wherein mitochondria are transferred from a donor cell to a recipient cell, significantly influences the function and fate of the recipient cell^16^-^20^.

**Figure 1:**
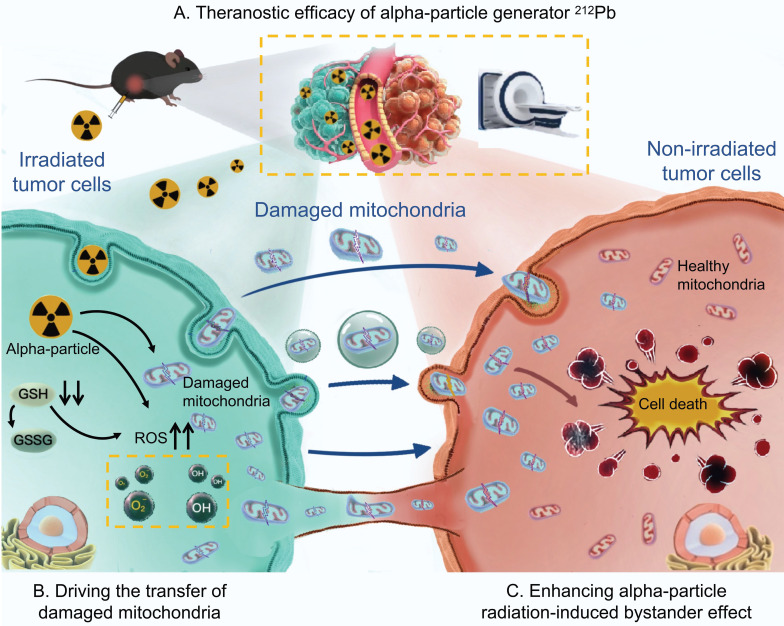
Graphical abstract. Theranostic efficacy of alpha-particle generator ^212^Pb and role of damaged mitochondrial transfer in alpha-particle radiation-induced bystander effect.

In this study, we aim to develop hydrogel nanoparticles (HNPs) loaded with the alpha-particle generator ^212^Pb to explore its theranostic potential. Additionally, we would like to elucidate the critical role of damaged mitochondrial transfer in the alpha-particle radiation-induced bystander effect (Figure 1).

## Materials and Methods

### Materials

Phosphate buffered saline (PBS), fetal bovine serum (FBS), dulbecco's modified eagle medium (DMEM), and a cell membrane protein extraction kit were purchased from Biosharp (Beijing, China). Calcein/Propidium Iodide (PI) cell viability/cytotoxicity assay kit, reduced glutathione (GSH) and oxidized glutathione disulfide (GSSG) assay kits, mitochondrial membrane potential assay kit with JC-1, and the Annexin V-Fluorescein isothiocyanate (FITC) apoptosis assay kit were obtained from Beyotime Biotechnology Co., Ltd. (Shanghai, China). MitoTracker™ Red FM were purchased from Thermo Fisher (MA, USA). Rotenone was purchased from Sigma-Aldrich (St. Louis, USA).

### Instrument

The hydrodynamic diameter and Zeta potential of the nanoparticles were analyzed by the Malvern Zetasizer Nano ZS. Transmission electron microscopy (TEM) images were taken by FEI-Tecnai12. X-ray spectroscopy (EDS) mapping elemental analysis was performed at JEM-F200 (URP). Fluorescence imaging was performed by Carl Zeiss LSM900 confocal microscope and Nikon inverted fluorescence microscope. Absorbance and chemiluminescence were measured using the SpectraMax iD5 Multi-Mode Microplate Readers (Molecular Devices, USA). Flow cytometry was performed on a BD Fortessa flow cytometer. Imaging of ^212^Pb was performed using single-photon emission-computed tomography (SPECT/CT) ((InliView-3000B, Novel Medical)).

### Separation of ^212^Pb and ^212^Bi

^212^Pb and^ 212^Bi were obtained from a^ 224^Ra-^212^Pb/^212^Bi generator prepared in our laboratory. After isolating ^224^Ra from^ 228^Th, it was absorbed on a AG50W×8 cation resin column. ^212^Pb and ^212^Bi was separated from ^224^Ra by eluting the column with 2 _M_ HCl and 0.5 _M_ HCl respectively.^ 212^Pb was further purified by anion exchange resin AGMP-1M and eluted by 0.1 _M_ HNO_3_ and it is possible that^ 212^Pb exists in the form of Pb^2+^. Before labelling, the pH value was adjusted by NH_4_OH to 6.5-7.5. ^212^Bi was purified by a DGA resin cartridge. After loading ^212^Bi onto a DGA resin column, it was finally eluted by 50 m_M_ ammonium citrate solution with a pH value of 7.0±0.5 and it is reasonable that ^212^Bi is in the chemical form of bismuth citrate in solution. The activity of ^212^Bi and ^212^Pb was determined by liquid scintillation counter (LSC) after the equilibrium has been achieved between ^212^Bi and ^212^Pb 21. The purity of^ 212^Pb and^ 212^Bi was confirmed by measuring the decay half-life using LSC. Radionuclides with half-lives not exceeding 5% of the reference value were applied in all the experiment. For ^212^Pb, the purity was further determined by measuring the activity of^ 224^Ra after 12-15 half-lives of ^212^Pb and the results indicated that the breakthrough of ^224^Ra is less than 0.03%.

### Synthesis of HNPs and Pb/ ^212^Pb-HNPs

The material was synthesized following the procedure previously reported 22 Typically, Span 80 (300 mg) and Tween 80 (100 mg) were dissolved in 15 mL of cyclohexane. Subsequently, a mixed solution containing 250 μL of PEGDA and 550 μL of water was added. The mixture was transformed into an emulsion by ultrasonication for 2 min. Next, 100 μL of the photoinitiator 2-hydroxy-2-methylpropiophenone was added to the emulsion. The reaction was carried out under magnetic stirring at 800 rpm using a 365 nm longwave UV lamp for 4 h. The products were then collected by centrifugation at 12,000 rpm and washed three times with ethanol and water. Finally, Pb/^212^Pb and water were added to the HNPs, ensuring that the total volume of the system was controlled at 3 mL. The mixture was vigorously stirred for 1 h, followed by centrifugation and washed three times to collect the precipitate.

### Characterization of HNPs and Pb-HNPs

The size distribution and surface zeta potential of HNPs and Pb-HNPs were evaluated by dynamic light scattering (DLS). The morphology of HNPs and Pb-HNPs was characterized by TEM. the mapping images and EDS showed a uniform distribution of C, N, O and Pb elements over the entire architecture of the Pb-HNPs.

### Radiolabeling and labeling product stability assays

The purified ^212^Pb-HNPs was co-incubated with PBS and FBS (100 μL for each) at 37 ℃ for 1 h, 6 h, 12 h and 24 h. The labeling rate and stability at each time point were determined by LSC.

### Cellular uptake of ^212^Pb-HNPs

LLC cells were incubated in 24-well plates at a density of 1×10^5^/mL/well for 24 h. For cell uptake experiment at different time points, LLC cells were treated with 0.6 kBq/mL ^212^Pb-HNPs or ^212^Pb, and incubated for 0.5 h, 1 h and 2 h. The culture medium was removed post incubation and cells were washed twice with PBS. Cells were lysed with 1_ M_ sodium hydroxide solution and the cell components were collected. Finally, the radioactivity of cells and culture medium were counted by LSC.

### *In vitro* cytotoxicity analysis

Cell viability was measured by the CCK-8 method. LLC cells were seeded into 96-well plates at a density of 5×10^3^ cells per well and cultured in DMEM containing 10% FBS (100 μL) for 24 h. The old medium was removed, and the cells were treated with serum-free DMEM containing different concentrations of ^212^Pb, ^212^Pb-HNPs, ^212^Bi, ICCM, mitochondria or others. Besides, LLC cells were treated with 2 μ_M_ rotenone, an inhibitor of mitochondrial respiratory chain, for 1 h prior to irradiation, then immediately washed with PBS before replacing with fresh media. After 24 h or other specified time points, the medium was sucked out, replaced with fresh DMEM, and 10 μL CCK-8 solution was added to each well. Then the absorbance was measured at 450 nm by a microplate reader, and the cell survival rate was calculated.

### Detection of intracellular GSH and GSSG levels

The ability of ^212^Pb-HNPs to deplete GSH was assessed using GSH and GSSG assay kits. LLC cells were seeded in 6-well plates at a density of 5×10^5^ cells per well and cultured overnight to allow adherence. The cells were then co-incubated with HNPs (0.35 mg/mL), ^212^Pb (0.7 kBq/mL), and ^212^Pb-HNPs (0.7 kBq/mL) for 24 hours. Following incubation, the cells were washed once with PBS buffer and collected by centrifugation. According to the assay kit instructions, a protein removal reagent was added to the collected cells, which were then thoroughly vortexed and subjected to two rapid freeze-thaw cycles. The supernatant was collected after centrifugation for 10 minutes at 4°C. One portion of the supernatant was used to determine total GSH, while the other portion was used to determine GSSG content after removing GSH. Absorbance at 412 nm was measured using a microplate reader, and total GSH and GSSG were quantified by plotting a standard curve. Reduced GSH was calculated by subtracting twice the amount of GSSG from total GSH.

### Analysis of intracellular ROS generation

The ability of drugs to induce intracellular ROS production was assessed by measuring the fluorescence changes resulting from the oxidation of the 2'-7'-dichlorodihydrofluorescein diacetate (DCFH-DA) probe. LLC cells were cultured in 6-well plates at a density of 5×10^5^ cells per well for 24 hours. Subsequently, the cells were treated with HNPs (0.35 mg/mL), ^212^Pb (0.7 kBq/mL) and ^212^Pb-HNPs (0.7 kBq/mL) for an additional 24 hours. After treatment, the cells were collected and resuspended in diluted DCFH-DA solution, then incubated at 37°C for staining. The cells were washed three times with serum-free DMEM to thoroughly remove any DCFH-DA that had not entered the cells. Finally, the fluorescence intensity was analyzed by flow cytometry to quantify the intracellular ROS levels, and the data were processed using FlowJo software.

### Isolation of mitochondria

When the cell density reaches 70%-80%, irradiate with ^212^Bi for 24 h. The cell culture medium was collected and centrifuged at 600 g for 10 min at 4°C. The supernatant was transferred to a new centrifuge tube, discarding the pellet. Subsequently, the supernatant was centrifuged at 1200 g for 10 min at 4°C. Discard the pellet, and transfer the supernatant to another new centrifuge tube. Finally, centrifuge at 8000 g for 15-30 min at 4°C. Carefully remove the supernatant, and the resulting pellet contains the extracellular mitochondria 23-25.

### Mitochondrial membrane potential measurement

To monitor mitochondrial health, JC-1 dye was used to assess mitochondrial membrane potential. LLC cells subjected to different treatments were incubated with JC-1 (5 μ_M_) for 30 min at 37°C. JC-1 dye accumulates in mitochondria in a potential-dependent manner, indicated by a fluorescence emission shift from green (Ex 485 nm/Em 516 nm) to red (Ex 579 nm/Em 599 nm). Mitochondrial membrane potential was determined using flow cytometry or confocal microscopy.

### Measurement of mitochondrial morphology

The transmission electron microscopy was used to evaluate the capability of ^212^Pb-HNPs to damage mitochondria and to demonstrate the presence of damaged mitochondria in ICCM. Samples were initially fixed in 2.5% glutaraldehyde and subsequently post-fixed in buffered 1% osmium tetroxide. They were then dehydrated through a graded series of acetone and embedded in Epon resin. Ninety-nanometer-thin sections were cut using a Leica EM UC6 ultramicrotome. The sections were stained with a saturated solution of uranyl acetate and lead citrate. Images were captured using a Hitachi TEM system. Mitochondrial morphology was also evaluated by MitoTracker™ Red FM, following the manufacturer's instructions. Cells were cultured on different substrates for 24 h and then incubated with MitoTracker™ Red dye (200 n_M_) for 20 min at 37 °C. Images of labeled mitochondria were captured using confocal microscopy.

### Animal model construction

The C57BL/6 mice (female, 6 weeks old) used in the experiment were provided by the animal center of the China Institute for Radiation Protection and were raised in a specific pathogen-free (SPF) environment. All animal experiments were reviewed and approved by the Experimental Animal Welfare Ethics Committee of Shanghai Tenth People's Hospital. To construct a tumor-bearing mouse model, LLC cells in a good logarithmic growth state were used. A cell suspension containing 5×10^6^ cells in PBS buffer (100 μL) was prepared and injected subcutaneously into the back of each mouse.

### SPECT/CT imaging

When the tumor volume of tumor-bearing mice reached about 500 mm^3^, the mice were anesthetized using 2% isoflurane for imaging. SPECT/CT images with ^212^Pb or ^212^Pb-HNPs (74 kBq per mouse, intratumor injection) were obtained on a SPECT/CT device equipped with a high-energy collimator detecting the ^212^Pb characteristic γ-rays (238 keV, 43.6%). Specifically, a window width of 20% was utilized to ensure accurate capture of the relevant gamma emissions. The image reconstruction was conducted using the Ordered Subset Expectation Maximization (OSEM) algorithm, which is known for its efficiency in handling emission tomography data. Post-reconstruction, median filtering was applied to enhance image quality by reducing noise while preserving edges. The reconstructed images were configured into a 60×60 matrix with a pixel size of 0.7 mm, ensuring adequate resolution for detailed analysis. The slice thickness was also set at 0.7 mm to provide uniformity across slices. The iterative reconstruction process involved 40 iterations, which were crucial for achieving optimal convergence and image clarity. The scan covered a length of 54 mm, segmented into multiple acquisitions with a total bed step count of 88 steps. Each step incorporated an angular increment of 3.0 degrees to facilitate comprehensive volumetric data collection. Imaging sessions were conducted at 30 minutes of each scan at different time points (30 minutes and 2 hours post-intratumor injection).

### *In vivo* RNA‑seq analysis

When the tumor grew to 50 mm^3^, the tumor-bearing mice were randomly divided into 2 groups (n = 3 per group). it's worth mentioning that we could only perform injections in one mouse each for the control and experimental groups on the same day, followed by three repeated experiments. The day of grouping was designated as Day 1, marking the commencement of drug administration. The control group received 100 μL of PBS buffer, while the treatment group was administered ^212^Pb-HNPs 148 kBq) via intratumor injection. To investigate the microenvironment of residual tumors post-^212^Pb irradiation, tumor tissues were harvested from mice 7 days after resection for RNA sequencing. RNA extraction, quality control, library construction, and sequencing were performed according to the company's standard procedures. The featureCounts (v1.5.0-p3) and DESeq2 (v1.16.1) software were used to quantify gene expression and perform differential expression analysis between the two comparison groups, respectively. Gene Ontology (GO) enrichment analysis and the statistical enrichment of differentially expressed genes in the Kyoto Encyclopedia of Genes and Genomes (KEGG) pathways were conducted using clusterProfiler (v3.4.4).

### Live/dead cell staining assay

The *in vitro* efficacy of ICCM, mitochondria and others were further evaluated by live/dead staining. LLC cells were seeded into 24-well plates (1×10^5^ cells per well) and incubated overnight. The cells were then treated with appropriate doses of ICCM, mitochondria, and other substances, with an untreated group serving as the control. After 24 hours, the cells were stained with Calcein-AM and PI. Following a 1-hour incubation at 37°C, the staining effects were observed using a fluorescence microscope.

### Statistical approach

All data were expressed as mean values ± standard deviation (SD). Statistical analysis was implemented by using the Student's t-test between two groups and analysis of variance (ANOVA) when more than two groups. *p* < 0.05 is considered statistically significant.

## Results

### Synthesis and characterization of HNPs and Pb-HNPs

**Figure 2:**
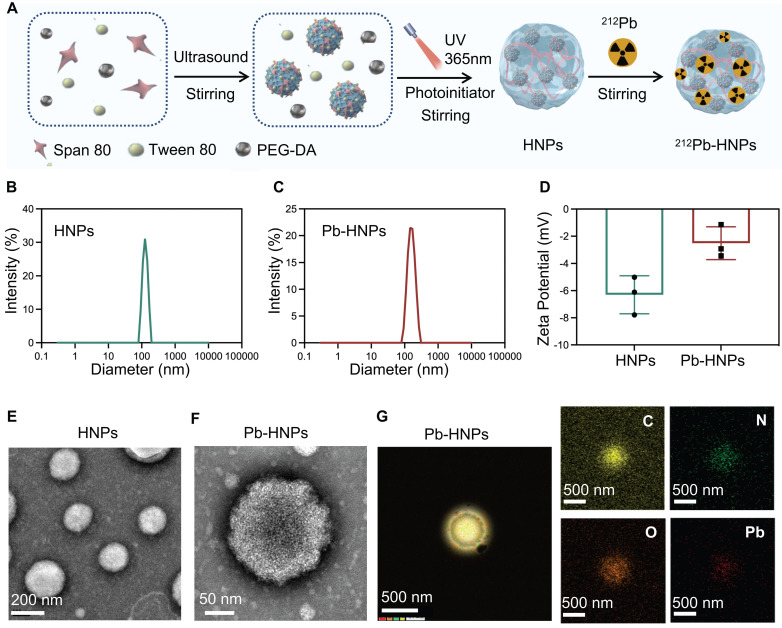
Synthesis and Characterization of HNPs and Pb-HNPs. A) Schematic illustration of the formation of HNPs and ^212^Pb-HNPs. B and C) DLS of HNPs and Pb-HNPs. D) Zeta potential of HNPs and Pb-HNPs. E and F) TEM image of HNPs and Pb-HNPs. G) The EDS mapping of C, N, O and Pb of prepared Pb-HNPs.

The synthesis process of HNPs and ^212^Pb-HNPs was illustrated in Figure 2A. DLS data indicated a slight reduction in both particle size and zeta potential of Pb-HNPs compared to HNPs (Figure 2B-D). The morphology of the synthesized HNPs and Pb-HNPs was characterized using TEM (Figure 2E, F). The images revealed that the size of HNPs ranged from 100 to 200 nm, exhibiting a spherical structure. Besides, upon doping with lead, the morphology remained unchanged. The mapping images and EDS showed a uniform distribution of C, N, O and Pb elements over the entire architecture of the Pb-HNPs (Figure 2G).

### Characterization and antitumor properties of ^212^Pb-HNPs* in vitro*

**Figure 3:**
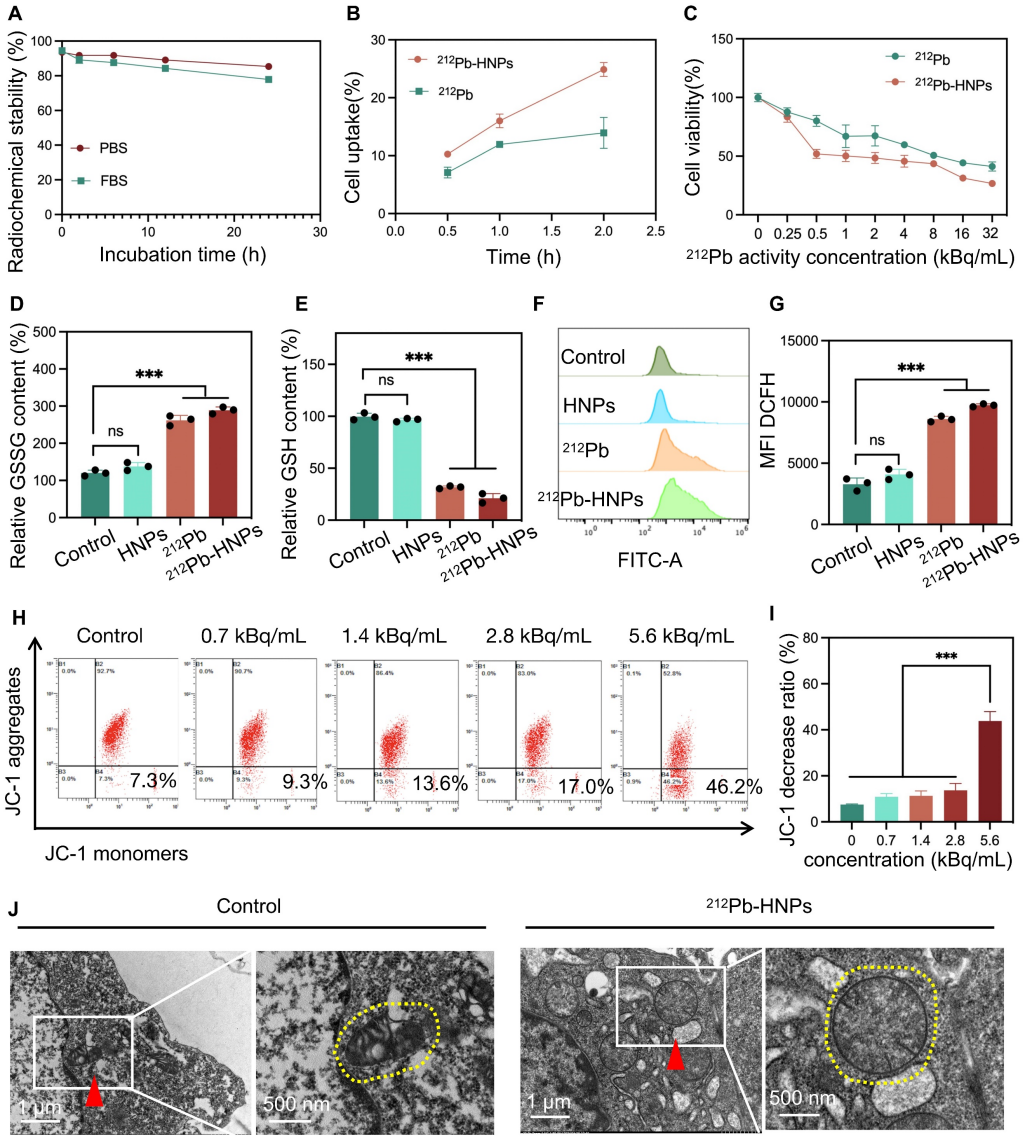
Characterization and antitumor properties of ^212^Pb-HNPs* in vitro*. A) The stability analysis of ^212^Pb-HNPs incubated in PBS and FBS (37℃) for 1 h, 6 h, 12 h and 24 h. B) The uptake comparison of^ 212^Pb and^ 212^Pb-HNPs in LLC cells. C) The cytotoxic effect of ^212^Pb and ^212^Pb-HNPs with gradient concentration on LLC cells for 24 h was evaluated by CCK-8 assays (n=4 per group). D and E) GSSG and GSH levels in LLC cells after different treatments. F and G) Fluorescence images and the corresponding mean fluorescence intensity (MFI) of ROS in LLC cells stained with DCFH-DA after various treatments. H and I) Flow cytometry patterns of JC-1 decrease in LLC cells after different treatments. J) Representative pictures of mitochondrial morphology observed under transmission electron microscope (red triangle, left), with a high magnification (yellow dashed circle, right).

The radiolabeled ^212^Pb-HNPs exhibited a radiochemical purity of up to 95% and showed prolonged radiolabeling stability under the conditions of PBS and FBS at 37℃. The results demonstrated that the residual radiolabeling stability could nearly reach 80% after 24 hours, which is sufficient for killing tumors (Figure 3A). First, the cellular uptake efficiency of ^212^Pb-HNPs was validated. The results indicated that ^212^Pb-HNPs exhibited a higher cellular uptake rate compared to free ^212^Pb (Figure 3B). This enhanced uptake is likely due to the material-bound radionuclides precipitating around the cells, whereas free radionuclides may remain suspended in the cell culture medium. We employed the CCK-8 assay to assess the impact of ^212^Pb-HNPs on the proliferation of LLC cells (Figure 3C). Due to the short range of alpha-particles, which is only 5-10 cell diameters, cells treated with the radionuclide-loaded nanomaterials are more susceptible to radiation-induced damage compared to those exposed to free radionuclides. As demonstrated in Figures 3D and 3E, ^212^Pb-HNPs exhibited the most pronounced GSH depletion ability, while other groups also showed a certain degree of GSH depletion. To further validate the ROS generation capability of ^212^Pb-HNPs, we employed DCFH-DA as a probe and conducted flow cytometry analysis. Upon cellular internalization, DCFH-DA undergoes hydrolysis catalyzed by esteraze, resulting in the formation of DCFH. Subsequently, ROS oxidize the initially non-fluorescent DCFH, converting it into fluorescent DCF. The results revealed that the ROS levels generated by ^212^Pb-HNPs were significantly higher compared to HNPs and ^212^Pb alone (Figures 3F and 3G), indicating that this novel radiopharmaceutical exhibits the strongest ability to induce ROS production. Additionally, to investigate the effects of the alpha-particle generator ^212^Pb on the mitochondria of irradiated cells, JC-1 staining was employed to assess the decline in mitochondrial membrane potential. As shown in Figure 3H and I, the extent of mitochondrial membrane potential decrease became more pronounced with increasing radiation doses, indicating more severe mitochondrial damage. Furthermore, TEM was used to observe the morphology of mitochondria in irradiated tumor cells, compared to the control group, the mitochondria in ^212^Pb-irradiated tumor cells exhibited a morphological shift from their typical ovoid shape to a more rounded appearance (Figure 3J). These results suggest that the alpha-emitter generator ^212^Pb induces mitochondrial damage in irradiated tumor cells.

### The potential theranostic efficacy of ^212^Pb-HNPs *in vivo*

**Figure 4:**
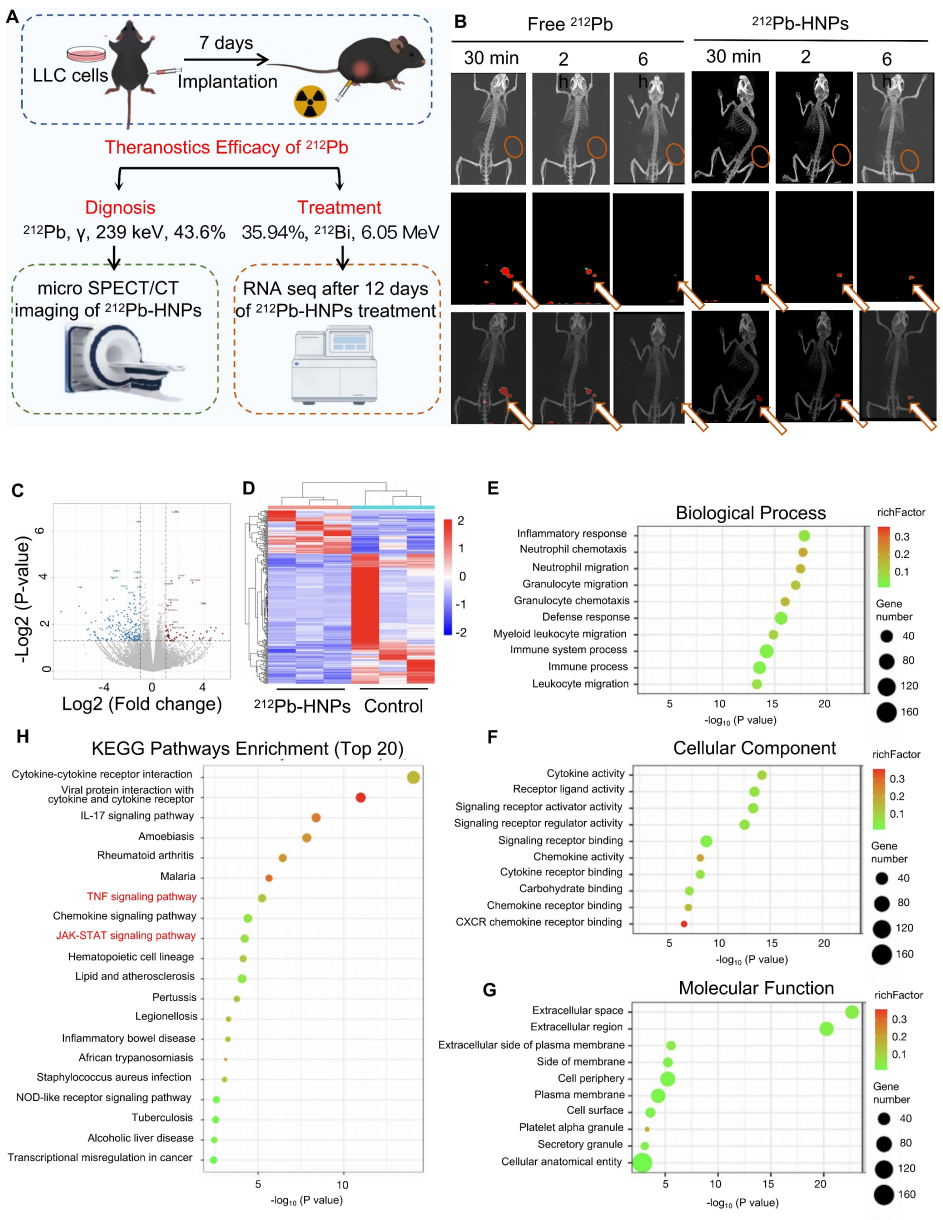
The potential theranostic efficacy of ^212^Pb-HNPs *in vivo.* A) Schematic representation of the experimental design for the subsequent study. B) Representative SPECT/CT imaging of LLC tumor-bearing mice post-intratumor injection of ^212^Pb or ^212^Pb-HNPs at different time points. C) Volcano plots showing the DEGs between the ^212^Pb-HNPs and control groups. D) Heatmap of DEGs between the ^212^Pb-HNPs and control groups. E) Biological process analysis. F) Cellular component analysis. G) Molecular function analysis. H) KEGG pathways enrichment analysis (TOP 20).

Based on these findings, it is suggested that ^212^Pb-HNPs holds substantial promise for *in vivo* tumor therapy. To further investigate the theranostics potential of^ 212^Pb, subcutaneous LLC xenograft tumor models were established in mice (Figure 4A). Small animal SPECT/CT was employed to validate the imaging capabilities of ^212^Pb. Upon intratumoral injection of ^212^Pb-HNPs, radioactivity uptake at the tumor site was observed even at 6 h post-injection, whereas there was little radioactivity in free ^212^Pb (Figure 4B). This result suggests that ^212^Pb-HNPs exhibit prolonged retention in the tumor region. Although we observed accumulation of free ^212^Pb in the bladder of mice at 30 minutes post-injection, we did not perform biodistribution studies by sacrificing the mice at different time points following imaging. In this current study, our conclusion is limited to demonstrating the imaging potential of ^212^Pb. Future studies are necessary to investigate the pharmacokinetics of ^212^Pb.

Due to the limited yield of ^212^Pb, we were unable to meet the demand for animal experiments. We could only perform injections in one mouse each for the control and experimental groups on the same day, followed by three repeated experiments. Based on the limitations of radionuclide yield, we divided the subjects into three groups: control group, 74 kBq group, and 148 kBq group. Tumor development was monitored according to the administration schedule. Regrettably, due to the small therapeutic doses administered, although partial tumor regression was observed in the experimental groups, the overall therapeutic efficacy was not satisfactory. Therefore, we did not present the data. However, RNA sequencing was performed on tumor samples from the mice after 7 days treatment (Control group: PBS; Treatment group: 148 kBq ^212^Pb-HNPs i.t.) to analyze the related results. The findings suggested that ^212^Pb-HNPs could induce oxidative stress in the tumor tissues (Figure 4C-H). These findings are preliminary but significant as they provide initial insights into the molecular mechanisms of oxidative stress induction by ^212^Pb-HNPs. Combined with results from ROS measurement and GSH determination at the cellular level, these findings suggest the therapeutic potential of ^212^Pb.

### Radiation-induced bystander effect triggered by alpha-particle ^212^Bi

**Figure 5:**
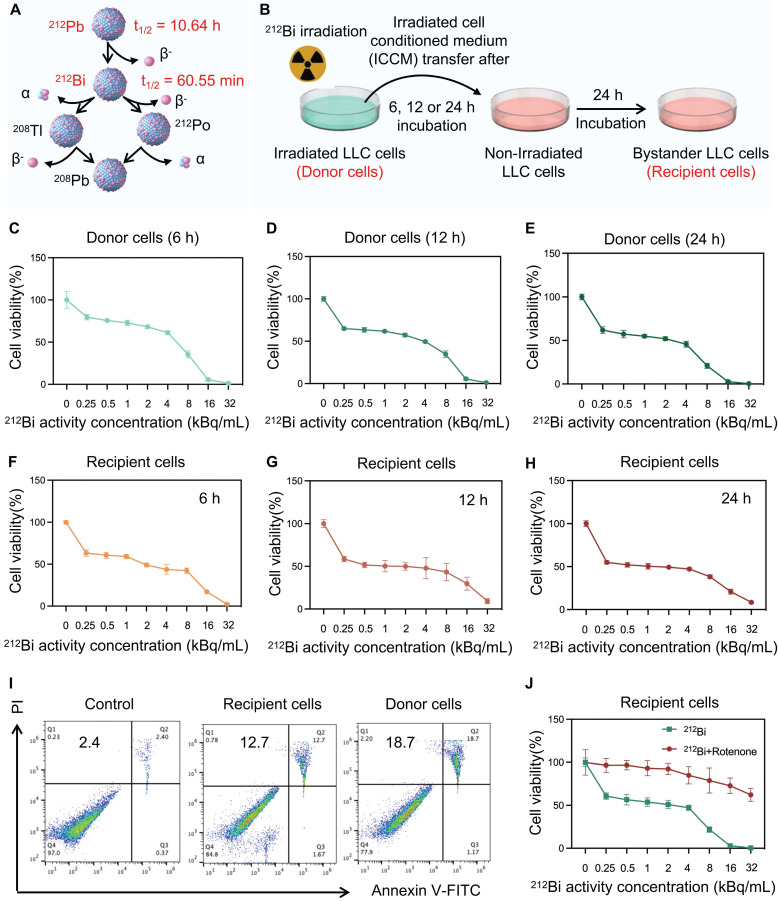
Radiation-induced bystander effect triggered by alpha-particle ^212^Bi. A) Decay scheme of alpha-particle generator ^212^Pb. B) Schematic representation of the experimental design for the subsequent cytotoxic effect study. C, D, and E) The cytotoxic effect of ^212^Bi with gradient concentration on donor cells for 6 h(C), 12h (D), 24 h (E), respectively (n=5). F, G, and H) The cytotoxic effect of supernatant from donor cells after 6 h (F), 12 h (G), and 24 h (H) ^212^Bi-irradiation with gradient concentration on recipient cells for 24 h (n=5). I) Flow cytometry patterns of recipient cells and donor cells stained with annexin Ⅴ-FITC and propidium iodide (PI) for determining the apoptosis level. J) The cytotoxic effect of supernatant after 24 h ^212^Bi-irradiation with gradient concentration on rotenone-pretreated recipient cells for 24 h.

To further validate the radiobiological effects of ^212^Pb on non-targeted tumor cells, the ^212^Pb-induced bystander effect was investigated. However, due to the 10.6 h half-life of^ 212^Pb, it was challenging to completely eliminate the radiation impact of residual ^212^Pb in the supernatant. Although ^212^Pb properties are different from its daughter nuclide^ 212^Bi, under specific conditions, they can be used to simulate and validate radiation-induced cellular damage effectively. Therefore, we chose to use ^212^Bi due to its immediate radiation effects which are easier to observe and record at the cellular level. This choice allowed us to gain preliminary insights into radiation-induced cellular damage mechanisms. Consequently, we employed ^212^Bi directly for cell-level validation (Figure 5A).

As Figure 5B shows, tumor cells were irradiated with ^212^Bi (donor cells) at a gradient dose, and the cell supernatant was extracted at 6 h, 12 h, and 24 h post-irradiation. The viability of these donor cells was subsequently examined (Figure C-E). This supernatant was then added to non-irradiated cells (bystander cells or recipient cells), and the activity of the bystander cells was monitored (Figure F-H). The results indicated varying degrees of cell death in the bystander cells, which exhibited a dose-dependent relationship. It is suggested that the presence of some intercellular communication factors in the supernatant affect the viability of the bystander cells. Furthermore, apoptosis assays were employed to further confirm the presence of alpha-particle RIBE (Figure 5I). Tumor cells were irradiated using a 6-well plate format. Each well received ^212^Bi at a radioactivity concentration of 3 kBq/mL. The chosen radioactivity concentration of 3 kBq/mL was determined based on preliminary dose-response studies to ensure an optimal balance between inducing measurable biological effects and maintaining cell viability. Given that the previous studies demonstrated that ^212^Pb-HNPs could induce ROS generation in tumor cells, we hypothesized that ROS might be a critical influencing factor in this context. To investigate this, we pretreated the bystander cells with rotenone, an ROS inhibitor, prior to exposing them to the supernatant from donor cells. The results showed a significant increase in the viability of the bystander cells. This suggests that ROS is one of the key factors involved (Figure 5J).

### Damaged mitochondrial transfer induced by ^212^Bi

**Figure 6:**
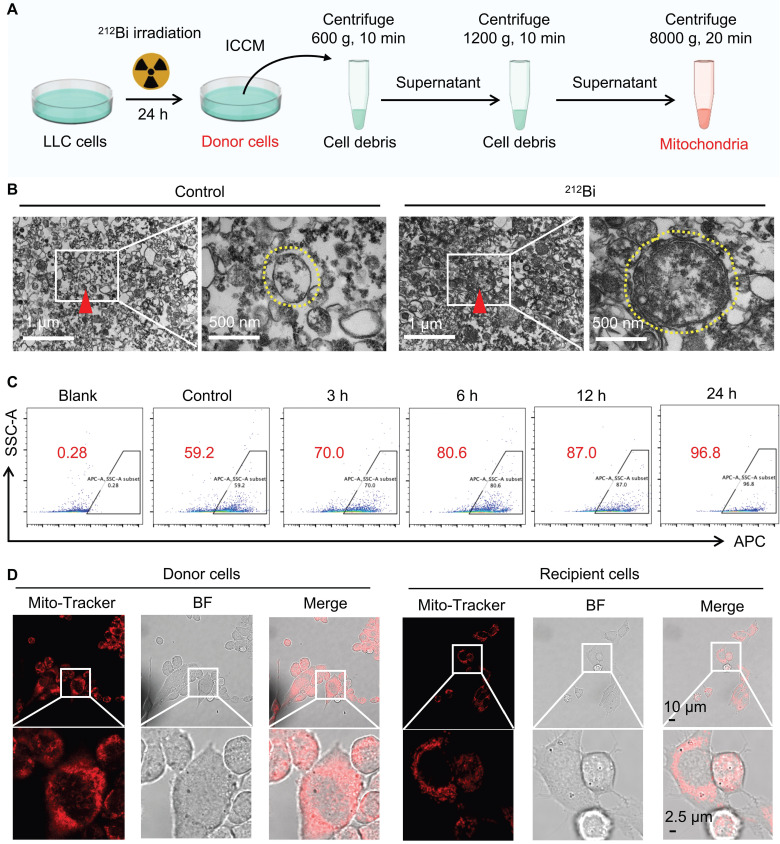
Damaged mitochondrial transfer induced by ^212^Bi. A) Outline the protocol for an isolation of mitochondria from irradiated cells. B) Transmission electron microscope demonstrating the free mitochondria (red triangle, left) in culture medium of LLC cells after different treatments, with a high magnification (yellow dashed circle, right). C) The number of free mitochondria in culture medium following ^212^Bi irradiation at various time intervals. D) Visualization of Mito-Tracker (red) in donor cells and recipient cells, indicating the free mitochondria in culture medium of donor can be transferred to recipient.

To further investigate whether alpha-particles can induce the transfer of damaged mitochondria, we employed gradient density centrifugation to isolate mitochondria from irradiated cell-conditioned media (ICCM) (Figure 6A). As shown in Figure 6B, TEM confirmed that this isolation method successfully extracted damaged mitochondria that had transferred into the ICCM. Flow cytometry analysis revealed a gradual increase in the number of damaged mitochondria with extended irradiation time (Figure 6C and Figure S1). To determine whether the damaged mitochondria in ICCM could enter non-irradiated tumor cells, we labeled the mitochondria of donor cells with MitoTracker™ Red FM and co-incubated the isolated mitochondria with recipient cells. Confocal microscopy revealed that the mitochondria from donor cells were marked in red and exhibited abnormal morphology, indicating mitochondrial damage. Interestingly, red-labeled mitochondria were also observed in bystander cells, suggesting that the mitochondria from irradiated donor cells could transfer to recipient cells (Figure 6D). These experimental results indicate that alpha-particle ^212^Bi can drive the transfer of damaged mitochondria.

### The transfer of damaged mitochondria results in alpha-particle radiation-induced bystander effect

**Figure 7:**
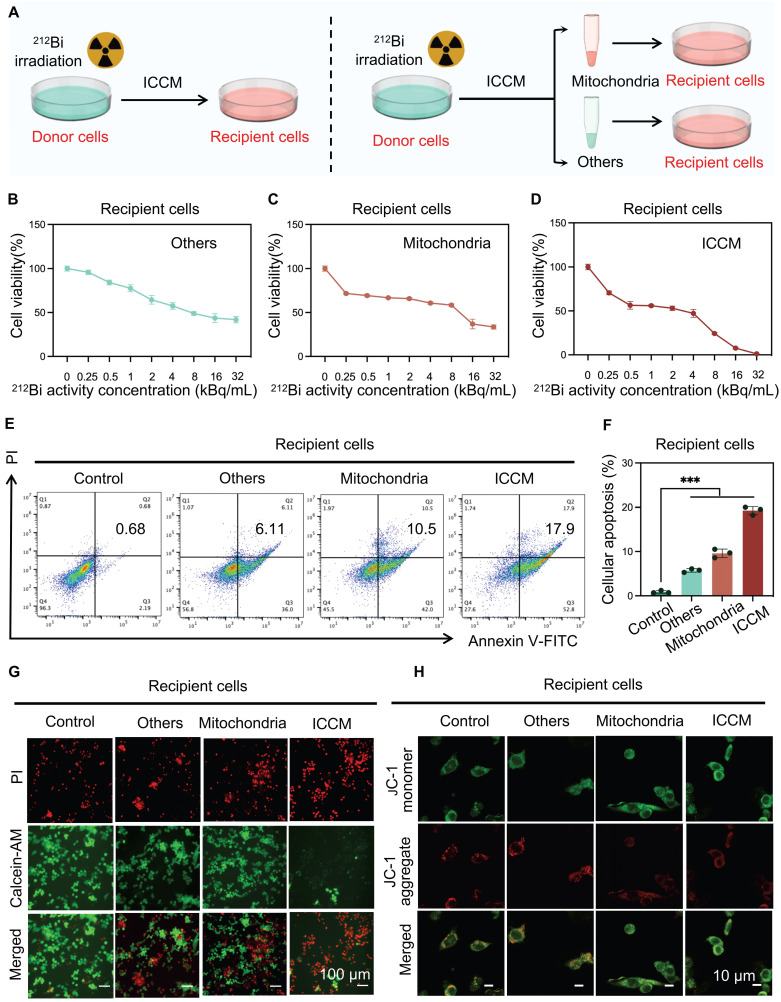
The transfer of damaged mitochondria results in alpha-particle radiation-induced bystander effect. A) Schematic representation of the experimental design for the subsequent study. B, C, and D) The cytotoxic effect of various treatments with gradient concentration on recipient cells for 24 h was evaluated by CCK-8 assays (n=5 per group). E and F) Flow cytometry patterns of recipient cells stained with annexin Ⅴ-FITC and PI after various treatments for determining the apoptosis level. G) Cytotoxicity of various treatments on recipient cells by using co-staining with PI and Calcein-AM. H) Visualization of JC-1 monomer (Green) and JC-1 aggregate (Red) in recipient cells after various treatments.

To further investigate the role of transferred damaged mitochondria in alpha-particle radiation-induced bystander effects, recipient cells were treated with the ICCM from ^212^Bi-irradiated cells, the mitochondria transferred into the ICCM, and other remaining substances, respectively, and observed the viability of the recipient cells (Figure 7A). CCK8 assay results indicated that the transferred damaged mitochondria significantly inhibited the viability of recipient cells, exhibiting a similar effect to that of ICCM (Figure 7B-D). Apoptosis assays and live/dead cell staining further confirmed the anti-tumor effects of transferred damaged mitochondria (Figure 7E-G). Additionally, confocal microscopy analysis of JC-1-stained recipient cell mitochondria revealed an increase in JC-1 monomers following treatment with damaged mitochondria, indicating a decrease in mitochondrial membrane potential and suggesting mitochondrial damage within the recipient cells (Figure 7H).

## Discussion

Due to the fact that alpha-particles exhibit unique clinical advantages such as "high energy, short range, hypoxia resistance, and ease of shielding." These properties highlight their tremendous potential in the precise treatment of tumors in nuclear medicine, suggesting they may drive significant shifts in therapeutic paradigms and concepts within the field. However, the therapeutic mechanisms of alpha-particles are more complex compared to beta-particles, and research on alpha-particles is still in its infancy globally 26. Numerous fundamental theoretical "uncharted territories" exist, posing key bottlenecks that hinder their clinical translation and application.

^212^pb is the current cutting-edge hotspot in the field of targeted nuclide therapy, and its decay releases the alpha-particle ^212^Bi with an energy of 6.05 MeV (half-life 60.6 min), which is often used as an *in vivo* alpha-particle generator. Compared with other long half-life alpha-particles (e.g. ^225^Ac,^ 223^Ra), ^212^Pb (half-life 10.6 hours) has a simpler and more efficient decay chain, which can rapidly decay and release alpha-particles, and the energy generated from the decay of this more 'high-dose-rate' alpha-particles can be rapidly deposited into the tumor, with off-target toxicity and daughter nuclide recoil. The off-target toxicity caused by daughter nucleus recoil and the toxic side effects caused by daughter nuclide redistribution are much smaller. In addition, the ^212^Pb decay process can release 238.6 keV γ-rays, which can be detected by SPECT/CT, and is expected to realize* in vivo* dynamic imaging, which is very promising for theranostics 27-30. In previous studies, ^203^Pb and ^212^ Pb are usually paired for the purpose of theranostics. Among them, ^203^Pb are usually administered at 18.5 MBq or 7.4 MBq per mouse, and there are also studies that used ^212^ Pb (7.4 MBq or 1.44 MBq) for *in vivo* imaging studies and 185 kBq or 100 kBq for postmortem biodistribution studies. All of these studies were administered at higher doses than we used. Due to the limitation of the technology, the maximum yield of the ^212^Pb that we can obtain at each time is only about 370 kBq at most. Besides, As the drug continues to decay, the dosage of each mouse (for free ^212^Pb or ^212^Pb-HNPs imaging) can only reach 74 kBq. Although the dose is very low, this study still demonstrates the great imaging potential of ^212^Pb after intratumor injection. In the future, we will aim to expand the yield of ^212^Pb and further improve the experiments on the theranostics of ^212^Pb. Besides, it is necessary to investigate the pharmacokinetics of ^212^Pb *in vivo*.

Currently, the therapeutic mechanisms of alpha-particles may be categorized into three main aspects. a) Nuclear DNA Damage Effect: Alpha-particles possess high linear energy transfer, which can induce irreparable double-strand breaks in nuclear DNA, thereby directly killing tumor cells. b) Immune Synergistic Effect: Alpha-particles could drive immunogenic cell death, with subsequently activating systemic anti-tumor immune responses, which can synergize with immunotherapies to enhance therapeutic efficacy. c) Bystander Effect: When only a small number of tumor cells are irradiated and damaged by alpha-particles, neighboring non-irradiated tumor cells can also exhibit damage effects 31. However, among various types of ionizing radiation, alpha-particles exhibit a unique pattern of heterogeneous ionization. The significant energy difference between regions of high ionization density and surrounding non-irradiated areas results in stronger biological signals that are more readily transmitted to non-irradiated cells 32-35. Consequently, the bystander effect induced by alpha-particles warrants particular attention in research and clinical applications.

In this study, we found that after treatment of tumor cells with the alpha-particle ^212^Bi, damaged mitochondria could be transferred to the extracellular environment. Upon further extraction and co-incubation of these damaged mitochondria with non-irradiated tumor cells, it was observed that the non-irradiated tumor cells could uptake the damaged mitochondria, resulting in reduced viability of these non-irradiated cells. This confirms that alpha-particles can drive the transfer of damaged mitochondria, which in turn can induce damage in non-irradiated tumor cells. Therefore, alpha-particles may mediate the bystander effect through the transfer of damaged mitochondria. Existing research has provided evidence that the transfer of damaged mitochondria can occur through three main pathways: tunneling nanotubes, extracellular vesicles, and free mitochondria 36. Once transferred to recipient cells via these mechanisms, the damaged mitochondria can significantly impact the function and fate of the recipient cells. Potential mechanisms by which transferred damaged mitochondria exert their effects include alterations in mitochondrial dynamics, increased genomic instability, and elevated oxidative stress 37-39. However, our study did not identify specific mechanisms related to alpha-particle. In future research, we intend to investigate these potential mechanisms more thoroughly. These mechanisms collectively contribute to the therapeutic potential of alpha-particles in oncology.

## Conclusion

This study validates the theranostics potential of the *in vivo* alpha-particle generator ²¹²Pb, with a particular focus on its ability to induce robust bystander effect. By elucidating the functional role of damaged mitochondrial transfer in the alpha-particle radiation-induced bystander effect, this research contributes to advancing the scientific understanding and clinical applications of targeted alpha therapy.

## Supplementary Material

Supplementary figure.

## Abbreviations

CCK-8: Cell Counting Kit-8
DLS: dynamic light scattering
EDS: energy dispersive spectrum
GO: Gene Ontology
HNPs: hydrogel nanoparticles
ICCM: irradiated cell-conditioned media
i.t.: intratumor
KEGG: Kyoto Encyclopedia of Genes and Genomes
LSC: liquid scintillation counter
MFI: mean fluorescence intensity
RIBE: radiation-induced bystander effect
ROS: reactive oxygen species
TAT: targeted alpha therapy
TEM: transmission electron microscopy

## References

[1] Siegel RL, Giaquinto AN, Jemal A (2024). Cancer statistics, 2024. CA Cancer J Clin.

[2] Feuerecker B, Kratochwil C, Ahmadzadehfar H, Morgenstern A, Eiber M, Herrmann K (2023). Clinical Translation of Targeted α-Therapy: An Evolution or a Revolution?. J Nucl Med.

[3] Zhang J QS, Yang M, Zhang X, Zhang S, Yu F (2023). Alpha-emitters and targeted alpha therapy in cancer treatment. iRadiology.

[4] Bauer D, Carter LM, Atmane MI, De Gregorio R, Michel A, Kaminsky S (2024). (212)Pb-Pretargeted Theranostics for Pancreatic Cancer. J Nucl Med.

[5] Michler E, Kästner D, Brogsitter C, Pretze M, Hartmann H, Freudenberg R (2024). First-in-human SPECT/CT imaging of [(212)Pb]Pb-VMT-α-NET in a patient with metastatic neuroendocrine tumor. Eur J Nucl Med Mol Imaging.

[6] Tu W, Dong C, Fu J, Pan Y, Kobayashi A, Furusawa Y (2019). Both irradiated and bystander effects link with DNA repair capacity and the linear energy transfer. Life Sci.

[7] Daguenet E, Louati S, Wozny AS, Vial N, Gras M, Guy JB (2020). Radiation-induced bystander and abscopal effects: important lessons from preclinical models. Br J Cancer.

[8] Burdak-Rothkamm S, Rothkamm K (2018). Radiation-induced bystander and systemic effects serve as a unifying model system for genotoxic stress responses. Mutat Res Rev Mutat Res.

[9] Nagasawa H, Little JB (1992). Induction of sister chromatid exchanges by extremely low doses of alpha-particles. Cancer Res.

[10] Miranda S, Correia M, Dias AG, Pestana A, Soares P, Nunes J (2020). Evaluation of the role of mitochondria in the non-targeted effects of ionizing radiation using cybrid cellular models. Sci Rep.

[11] Averbeck D (2023). Low-Dose Non-Targeted Effects and Mitochondrial Control. Int J Mol Sci.

[12] Ariyoshi K, Miura T, Kasai K, Fujishima Y, Nakata A, Yoshida M (2019). Radiation-Induced Bystander Effect is Mediated by Mitochondrial DNA in Exosome-Like Vesicles. Sci Rep.

[13] Missiroli S, Perrone M, Genovese I, Pinton P, Giorgi C (2020). Cancer metabolism and mitochondria: Finding novel mechanisms to fight tumours. EBioMedicine.

[14] Peng W, Wong YC, Krainc D (2020). Mitochondria-lysosome contacts regulate mitochondrial Ca(2+) dynamics via lysosomal TRPML1. Proc Natl Acad Sci U S A.

[15] Wang M, Smith K, Yu Q, Miller C, Singh K, Sen CK (2019). Mitochondrial connexin 43 in sex-dependent myocardial responses and estrogen-mediated cardiac protection following acute ischemia/reperfusion injury. Basic Res Cardiol.

[16] Chakrabarty RP, Chandel NS (2021). Mitochondria as Signaling Organelles Control Mammalian Stem Cell Fate. Cell Stem Cell.

[17] Liu Y, Fu T, Li G, Li B, Luo G, Li N (2023). Mitochondrial transfer between cell crosstalk - An emerging role in mitochondrial quality control. Ageing Res Rev.

[18] Borcherding N, Brestoff JR (2023). The power and potential of mitochondria transfer. Nature.

[19] Rajendran S, Harrison SH, Thomas RA, Tucker JD (2011). The role of mitochondria in the radiation-induced bystander effect in human lymphoblastoid cells. Radiat Res.

[20] Zhang H, Yu X, Ye J, Li H, Hu J, Tan Y (2023). Systematic investigation of mitochondrial transfer between cancer cells and T cells at single-cell resolution. Cancer Cell.

[21] Bergeron DE, Cessna JT, Fitzgerald RP, Laureano-Pérez L, Pibida L, Zimmerman BE (2022). Primary standardization of (212)Pb activity by liquid scintillation counting. Appl Radiat Isot.

[22] Chen X, Zhang S, Li J, Huang X, Ye H, Qiao X (2022). Influence of Elasticity of Hydrogel Nanoparticles on Their Tumor Delivery. Adv Sci (Weinh).

[23] Al Amir Dache Z, Otandault A, Tanos R, Pastor B, Meddeb R, Sanchez C (2020). Blood contains circulating cell-free respiratory competent mitochondria. Faseb j.

[24] Song X, Hu W, Yu H, Wang H, Zhao Y, Korngold R (2020). Existence of Circulating Mitochondria in Human and Animal Peripheral Blood. Int J Mol Sci.

[25] Hayakawa K, Esposito E, Wang X, Terasaki Y, Liu Y, Xing C (2016). Transfer of mitochondria from astrocytes to neurons after stroke. Nature.

[26] Boyd M, Ross SC, Dorrens J, Fullerton NE, Tan KW, Zalutsky MR (2006). Radiation-induced biologic bystander effect elicited in vitro by targeted radiopharmaceuticals labeled with alpha-, beta-, and auger electron-emitting radionuclides. J Nucl Med.

[27] Quelven I, Monteil J, Sage M, Saidi A, Mounier J, Bayout A (2020). (212)Pb α-Radioimmunotherapy Targeting CD38 in Multiple Myeloma: A Preclinical Study. J Nucl Med.

[28] Chapeau D, Koustoulidou S, Handula M, Beekman S, de Ridder C, Stuurman D (2023). [(212)Pb]Pb-eSOMA-01: A Promising Radioligand for Targeted Alpha Therapy of Neuroendocrine Tumors. Pharmaceuticals (Basel).

[29] Kvassheim M, Tornes AJK, Juzeniene A, Stokke C, Revheim MR (2023). Imaging of (212)Pb in mice with a clinical SPECT/CT. EJNMMI Phys.

[30] Li M, Sagastume EA, Lee D, McAlister D, DeGraffenreid AJ, Olewine KR (2020). (203/212)Pb Theranostic Radiopharmaceuticals for Image-guided Radionuclide Therapy for Cancer. Curr Med Chem.

[31] Yard BD, Gopal P, Bannik K, Siemeister G, Hagemann UB, Abazeed ME (2019). Cellular and Genetic Determinants of the Sensitivity of Cancer to α-Particle Irradiation. Cancer Res.

[32] Mukherjee S, Dutta A, Chakraborty A (2022). The cross-talk between Bax, Bcl2, caspases, and DNA damage in bystander HepG2 cells is regulated by γ-radiation dose and time of conditioned media transfer. Apoptosis.

[33] Ladjohounlou R, Lozza C, Pichard A, Constanzo J, Karam J, Le Fur P (2019). Drugs That Modify Cholesterol Metabolism Alter the p38/JNK-Mediated Targeted and Nontargeted Response to Alpha and Auger Radioimmunotherapy. Clin Cancer Res.

[34] Le M, McNeill FE, Seymour CB, Rusin A, Diamond K, Rainbow AJ (2018). Modulation of oxidative phosphorylation (OXPHOS) by radiation- induced biophotons. Environ Res.

[35] Dong C, Tu W, He M, Fu J, Kobayashi A, Konishi T (2020). Role of Endoplasmic Reticulum and Mitochondrion in Proton Microbeam Radiation-Induced Bystander Effect. Radiat Res.

[36] Al Amir Dache Z, Thierry AR (2023). Mitochondria-derived cell-to-cell communication. Cell Rep.

[37] Bock FJ, Tait SWG (2020). Mitochondria as multifaceted regulators of cell death. Nat Rev Mol Cell Biol.

[38] Kuo CL, Ponneri Babuharisankar A, Lin YC, Lien HW, Lo YK, Chou HY (2022). Mitochondrial oxidative stress in the tumor microenvironment and cancer immunoescape: foe or friend?. J Biomed Sci.

[39] Shimura T (2023). Mitochondrial Signaling Pathways Associated with DNA Damage Responses. Int J Mol Sci.

