# Supplementary material for: Role of damaged mitochondrial transfer in alpha-particle generator 212Pb radiation-induced bystander effect
Source: Theranostics. 2024 Oct 14;14(17):6768–82. doi: 10.7150/thno.101922 (PMC11519793; doi:10.7150/thno.101922)
Supplement: Supplementary file 1 — Supplementary figure. [file thnov14p6768s1.pdf]

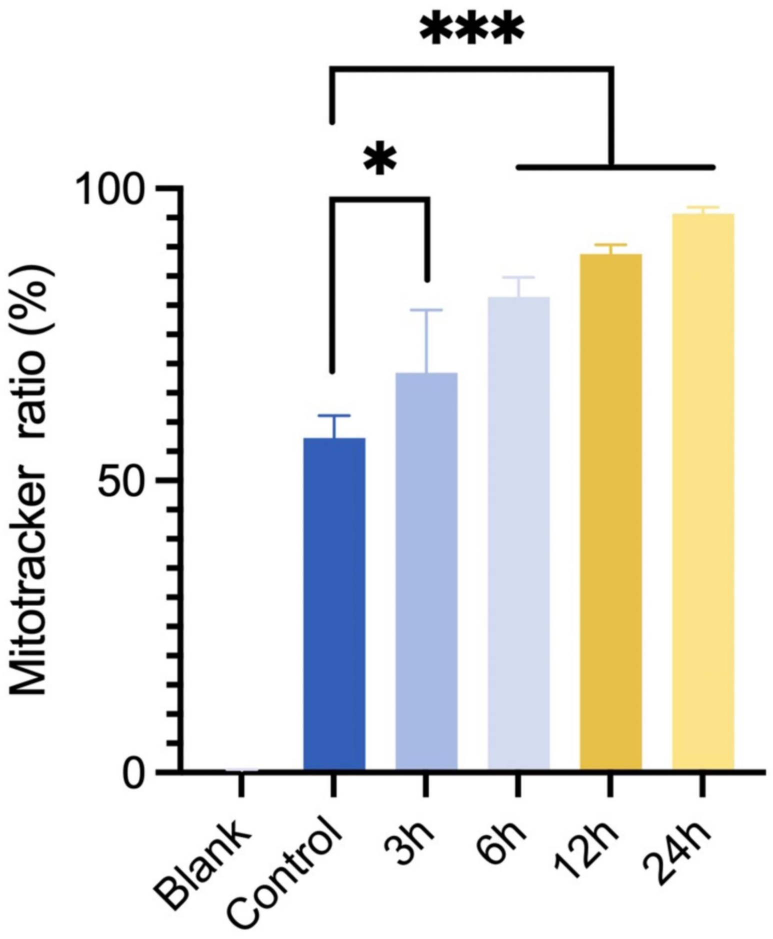

Figure S1. The number of free mitochondria in culture medium following ^{212}Bi irradiation at various time intervals.
